# Policies regulating retail environment to reduce tobacco availability: A scoping review

**DOI:** 10.3389/fpubh.2023.975065

**Published:** 2023-02-14

**Authors:** Raouf Alebshehy, Zara Asif, Melanie Boeckmann

**Affiliations:** ^1^School of Public Health, Bielefeld University, Bielefeld, Germany; ^2^Department for Health, University of Bath, Bath, United Kingdom; ^3^Department of Global Health and Social Medicine, King's College London, London, United Kingdom; ^4^Faculty of Human and Health Sciences, University of Bremen, Bremen, Germany

**Keywords:** tobacco retail environment, retail regulation, supply reduction of tobacco, availability reduction, tobacco sale

## Abstract

**Background:**

In 2005, the World Health Organization Framework Convention on Tobacco Control (WHO FCTC) entered into force. This treaty was developed in response to the global tobacco epidemic, and it includes measures to reduce both demand for and supply of tobacco. The measures related to demand reduction include raising tax, providing cessation services, promoting smoke free public places, banning advertising, and raising awareness. However, there are a limited number of measures for supply reduction, and these mainly include fighting illicit trade, banning sales to minors and providing alternatives to tobacco workers and growers. Unlike regulation of many other goods and services that have been subjected to retail restrictions, there is a lack of resources about restricting tobacco availability through regulation of tobacco retail environment. Considering the potential of retail environment regulations in reducing tobacco supply and consequently reducing tobacco use, this scoping review aims to identify relevant measures.

**Methods:**

This review examines interventions, policies, and legislations to regulate tobacco retail environment to reduce tobacco availability. This was done by searching the WHO FCTC and its Conference of Parties decisions, a gray literature search including tobacco control databases, a scoping communication with the Focal Points of the 182 WHO FCTC Parties, and a databases search in PubMed, EMBASE, Cochrane Library, Global Health, and Web of Science.

**Results:**

Themes of policies were identified to reduce tobacco availability by regulating retail environment: four WHO FCTC and twelve non-WHO FCTC policies. The WHO FCTC policies included requiring a license to sell tobacco, banning tobacco sale via vending machines, promoting economically alternative activities to individual sellers, and banning ways of sale that constitute a way of advertising, promotion, and sponsorships. The Non-WHO FCTC policies included banning tobacco home delivery, tray sale, tobacco retail outlets in or within a minimum distance from specific facilities, sale in specific retail outlets, and sale of tobacco or one or more of its products, in addition to restricting tobacco retail outlets per density of population and per geographic area, capping the tobacco amount allowed per purchase, limiting the number of hours or days in which tobacco can be sold, requiring a minimum distance between tobacco retailers, reducing tobacco products availability and proximity within a retail outlet, and restricting sale to government controlled outlets.

**Discussion and conclusion:**

Studies show the effects of regulation of the retail environment in influencing overall tobacco purchases, and there is evidence that having fewer retails reduces the level of impulse purchasing of cigarettes and tobacco goods. The measures covered by WHO FCTC are much more implemented than ones not covered by it. Although not all widely implemented, many themes of limiting tobacco availability by regulating tobacco retail environment are available. Further studies to explore such measures and the adoption of the effective ones under the WHO FCTC decisions, could possibly increase their implementation globally to reduce tobacco availability.

## 1. Introduction

Tobacco use results in the premature death of up to half of its users causing eight million deaths every year. The poisonous habit leads to major comorbidities included heart attacks and strokes and is considered a major risk factor for many types of cancers (1). In 2021, the projected global prevalence of tobacco use was estimated to be around 20.4% by 2025 among those aged 15 years and older (2). In 2003, the World Health Assembly adopted the World Health Organization Framework Convention on Tobacco Control (WHO FCTC), which came into force in 2005. This treaty was developed as an evidence-based treaty and became one of the most rapidly and widely embraced treaties in United Nations' history with 182 affiliated Parties (3).

The WHO FCTC includes measures relating to the reduction of both demand for and supply of tobacco. The measures related to demand reduction include raising tax, providing cessation services, promoting smoke free public places, banning advertising, and raising awareness. However, there are a limited number of measures for supply reduction and these mainly include fighting illicit trade, banning sales to minors, and providing alternatives to tobacco workers and growers (3).

Although limited, there are some implemented and evaluated policies related to tobacco supply reduction, especially in the retail environment, documented in published literature. Some of these include restrictions on the numbers, location, and opening hours of tobacco retail outlets; restricting the amount of tobacco purchased by smokers over a given time; and loss of retail license following breaches of any of the conditions. Tobacco control experts have suggested that such policies are the new frontier in tobacco control with huge potential role in fighting the epidemic (4). Unlike regulation of pharmaceuticals and many other goods and services that have been subjected to a wide variety of restrictions, there is a lack of information and resources about restricting tobacco availability through regulation of the tobacco retail environment (5).

Considering the potential of retail environment regulations in reducing tobacco supply and consequently reducing tobacco use, this review aims to identify the implemented and suggested policies to reduce tobacco availability by regulating retail environment. The epidemiological agent-host-environment model provides a useful framework for tobacco control (6). In this review, the conceptual framework is that policies and regulations (environment) aiming to reduce supply in the retail environment (agent) will help in decreasing tobacco use at population level (host).

This review aims to address the lack of information and resources about restricting tobacco availability by undertaking a systematic search to identify and compile all implemented or suggested interventions, policies, and legislations designed to regulate tobacco retail environment to reduce tobacco availability. Considering the purpose of this study and the lack of knowledge in this topic area, this study is decided to be a scoping review to investigate available information about policies for restricting tobacco availability through regulation of the tobacco retail environment. A scoping review is useful for examining emerging evidence and providing an overview or map of the evidence. Therefore, it is an ideal tool to determine the scope of the policies investigated in this study (7, 8).

## 2. Methods

This scoping review examines interventions, policies, and legislations (hereinafter referred to all as policies) designed to regulate tobacco retail environment to reduce tobacco availability. For this review, retail environment means the context that allows direct interaction between customer and seller to buy tobacco. This study identified policies that limit tobacco availability by regulating such an environment. This means that policies that limit tobacco availability by other means than regulating the retail environment were excluded such as policies related to fighting illicit trade in tobacco products.

Although not pubished, a protocol for this review was developed and agreed by the authors of the study. The protocol is reflected in the searches descriped within the methods section of this paper. The authors used the PRISMA-ScR checklist of Tricco et al. (8) for guidance in the writing of this paper (8).

### 2.1. Searches

This study underwent four phases of search to identify relevant policies. First, we searched for tobacco retail environment policies in the two international tobacco control treaties: (1) WHO FCTC, adoped in 2003, and the decisions of the Conference of the Parties taken at its nine sessions (2006–2021) (9) and (2) the Protocol to Eliminate Illicit Trade in Tobacco Products (the Protocol), adopted in 2012, and the decisions of its two sessions of the Meeting of the Parties (2018–2021) (10).

In May 2021, a scoping communication, Annex 1, was sent electronically to all Focal Points of the 182 Parties to the WHO FCTC, facilitated by the Secretariat of the WHO FCTC (Convention Secretariat), to elicit what policies are implemented or planned, within their jurisdiction, concerning reducing tobacco supply (availability) through retail environment regulations. We also tried to reach out to countries which are not Parties to the WHO FCTC through the regional WHO offices as the aim of the study is to scope all policies whether they follow the WHO FCTC or not.

A gray literature search was conducted that included (1) a search within the “tobaccocontrollaws.org” tobacco control legislation database, which is established and maintained by the International Legal Consortium of the Campaign for Tobacco-Free Kids (CTFK), and it contains tobacco control legislations of 211 countries, and it allows research by policy; (2) a search within the tobacco control implementation hub of the International Union Against Tuberculosis and Lung Disease which includes resources, evidence and case studies of tobacco control policies; and (3) reviewing documents shared within tobacco control community networks.

Lastly, based on the previously outlined search results, we identified key terms to be used within the scope of the review relating to tobacco, retail environments, policies, legislations, and regulations. These key terms were used to run searches in five databases: “PubMed”, “EMBASE”, “Cochrane Library”, “Global Health”, and “Web of Science”. The key terms run through the databases were:

– Tobacco and tobacco products: Tobacco OR Smok^*^ OR cigar^*^
– Policies to reduce tobacco availability: Regulat^*^ OR Polic^*^ OR Legislat^*^
– Retail environment: Retail^*^ OR outlet^*^ OR sale^*^ OR vending OR vendor OR store^*^ OR shop^*^

### 2.2. Inclusion and exclusion criteria

In the search within the documents of the WHO FCTC, the Protocol and the gray literature, all papers documenting or suggesting examples of interventions, policies, and legislations to regulate tobacco retail environment to reduce tobacco availability were included. The inclusion criteria consisted of papers that are published in English or Arabic; in any country; at any time; on tobacco retail environment regulation; and with no restriction on the type of study included. All full-text, peer reviewed articles including commentaries and editorials, as well as relevant gray literature were included. In addition to the inclusion criteria, the main exclusion criteria included studies focussing on regulations of tobacco demand reduction, or studies focusing on tobacco supply reduction by other means than regulating retail environment.

In the writing of this paper, the responses by WHO FCTC Focal Points relate to policies already identified by the search methods outlined in the gray literature search or the search within the WHO FCTC and the Protocol documents were excluded to avoid the burden of going through a validation process for policies already identified by other means. The peer-review and public availability criteria were not applied to the resources obtained from the WHO FCTC Focal Points.

In the database review, the inclusion criteria consisted of papers that are published in English or Arabic; in any country; at any time; on tobacco retail environment regulation; and with no restriction on the type of study included. All full-text, peer reviewed articles including commentaries and editorials, as well as relevant gray literature were included. In addition to the inclusion criteria, the main exclusion criteria included studies focussing on regulations of tobacco demand reduction, studies focusing on tobacco supply reduction by other means than regulating retail environment, or meaures already identified by other means of search.

### 2.3. Ethics

The Ethics Committee of Bielefeld University has reviewed the application of this review according to the ethical guidelines of the German Association of Psychology, which correspond to the guidelines of the American Psychological Association. The Ethics Committee of Bielefeld University approved the study as ethically appropriate.

### 2.4. Analysis

All policies identified through searching the documents of the WHO FCTC and the Protocol documents were assessed for inclusion in the review based on the predefined inclusion criteria. This review identified four policies covered by the international treaties. The policies are discussed in the results section (3.1.1–3.1.4), and listed in Table 1.

**Table 1:** Policies identified in the WHO FCTC and related documents.

**Document**	**Policy**
Article 15 of the WHO FCTC	– Requiring a license to sell tobacco
Guidelines for implementation of Article 13 of the WHO FCTC	– Banning tobacco sale *via* vending machines – Banning ways of sale that constitute a way of advertising, promotion, and sponsorships
Article 16 of the WHO FCTC	– Banning tobacco sale *via* vending machines
Article 17 of the WHO FCTC	– Promoting economically alternative activities to individual sellers
Article 6 of The Protocol	– Requiring a license to sell tobacco

The survey shared with the 182 WHO FCTC Focal Points was answered by 31 countries. Data was extracted from the answers and identified policies were described and presented in a tabular form and led to the identification of seven policies apart from the ones already covered by the WHO FCTC. New policies implemented or planned were included in the writing of the article as long as supporting evidence was provided by the WHO FCTC Focal Points or identified through gray literature, this led to the inclusion of responses from 19 countries in this review. The policies are discussed in the results section (3.2.1–3.2.7), and listed in Table 2.

**Table 2:** Policies identified from WHO FCTC Focal Points responses.

**Country**	**Policy**
France, Italy, South Korea	– Restricting tobacco retail outlets per density of population
Belgium, Costa Rica, South Korea	– Banning home delivery of tobacco
Guyana, Ireland	– Banning tray/ mobile tobacco sale
Andorra, Spain	– Capping the tobacco amount allowed per purchase
South Korea, Spain	– Requiring a minimum distance between tobacco retailers
Chile, France, Georgia, Guatemala, Honduras, Lebanon, Portugal, Saint Lucia, Saudi Arabia	– Banning tobacco retail outlets in or within a minimum distance from specific facilities
Czech Republic, France, Netherland, Saudi Arabia	– Banning tobacco sale in specific retail outlets

The CTFK database were searched under the category of sales restrictions, and this led to the identification of an additional policy, which is banning tobacco sale or one or more of tobacco products. Moreover, information from the CTFK database was used as source on number of countries implementing other four policies identified by the previous searchs. The policy identified is discussed in the results section (3.2.12), and all policies in this review that the CTFK database included information on are listed in Table 3.

**Table 3:** Policies identified from the CTFK database.

**Category**	**Policy**
Sales restrictions	– Requiring a license to sell tobacco – Banning tobacco sale *via* vending machines – Banning ways of sale that constitute a way of advertising, promotion, and sponsorships – Banning tobacco retail outlets in or within a minimum distance from specific facilities – Banning tobacco sale or one or more of tobacco products

All documents/ studies/ interventions identified through database searches or gray literature (the Union database and documents received through tobacco control networks) were assessed by two reviewers independently for inclusion in the review based on the predefined inclusion criteria. Any disagreement was resolved through mutual discussion. Data was extracted and identified policies were described and presented in a tabular form (11). Quality assessment and appraisal was not deemed necessary due to this being a scoping review (7).

**Figure 1:**
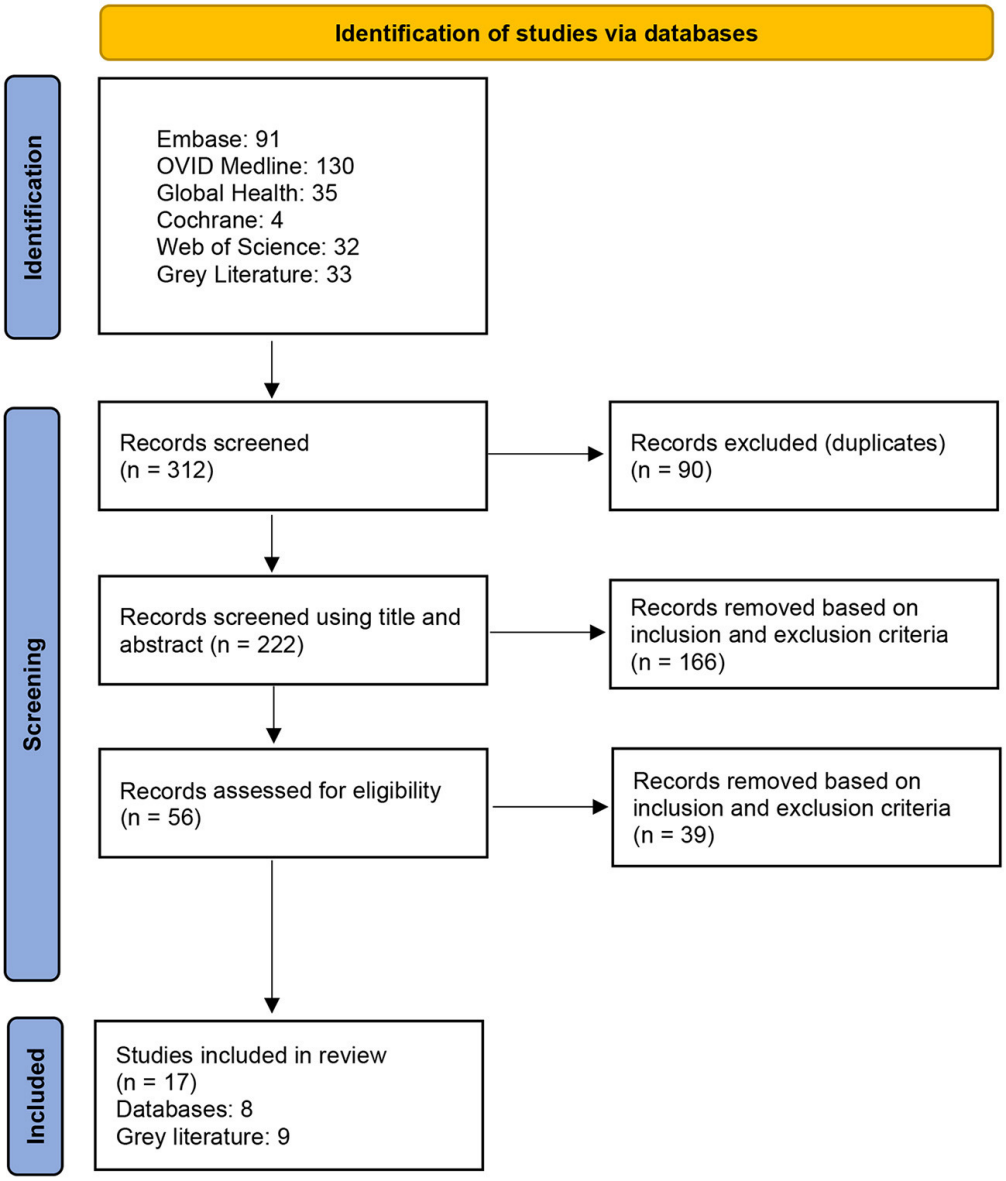
PRISMA flowchart.

Summary of the database and gray literature results can be found in the PRISMA diagram below for clarification of number of articles included and excluded based on the mentioned criteria. The gray literature and database search led to four additional policies being identified apart from the ones already identified by other searchs. The policy identified is discussed in the results section (3.2.8–3.2.11), and all policies in this review that the databases and gray literature included information on are listed in Table 4. A PRISMA flowchart shows the records of documents identified is presented in Figure 1.

**Table 4:** Policies identified by databases and gray literature search.

**Title**	**Authors**	**Setting**	**Study type**	**Policy**
**Papers identified through gray literature**				
Regulating the tobacco retail environment: beyond reducing sales to minors	Chapman S, Freeman B.	Australia	Review (communication piece)	• Requiring a license to sell tobacco
				• Government controlled outlets
				• Restricting tobacco retail outlets per density of population
				• Capping the tobacco amount allowed per purchase
Reducing the availability of tobacco products at retail: policy analysis	Tilson M.	Canada	Policy analysis	• Requiring a license to sell tobacco
				• Banning tobacco retail outlets in or within a minimum distance from specific facilities
				• Restricting tobacco retail outlets per geographic area
				• Requiring a minimum distance between tobacco retailers
				• Government controlled outlets
Reducing the Density and Number of Tobacco Retailers: Policy Solutions and Legal Issues	Ackerman A, Etow A, Bartel S, Ribisl K.	United States	Policy analysis	• Banning tobacco sale in specific retail outlets
				• Banning tobacco retail outlets in or within a minimum distance from specific facilities
				• Restricting tobacco retail outlets per geographic area
				• Requiring a minimum distance between tobacco retailers
				• Restricting tobacco retail outlets per density of population
				• Banning tobacco sale in specific retail outlets
A comparison of three policy approaches for tobacco retailer reduction	Myers A, Hall M, Isgett L, Ribisl K.	United States	Cross-sectional	• Banning tobacco sale in specific retail outlets
				• Banning tobacco retail outlets in or within a minimum distance from specific facilities
				• Requiring a minimum distance between tobacco retailers
Policy coherence, integration, and proportionality in tobacco control: Should tobacco sales be limited to government outlets?	Smith E, McDaniel P, Hiilamo H, Malone R.	United States	Policy analysis piece	• Government controlled outlets
Reducing Tobacco Retail Density in San Francisco: A Case Study	Bright Research Group	San Francisco, United States	Case study analysis	• Restricting tobacco retail outlets per geographic area
				• Requiring a minimum distance between tobacco retailers
				• Banning tobacco retail outlets in or within a minimum distance from specific facilities
				• Banning tobacco sale in specific retail outlets
				• Promoting economically alternative activities to individual sellers
Evaluating the impact and equity of a tobacco-free pharmacy law on retailer density in New York City neighborhoods	Giovenco D, Spillane T, Mauro C, Hernández D	New York City, United States	Cross-sectional	• Banning tobacco sale in specific retail outlets
Global review of tobacco product flavor policies	Erinoso O, Clegg Smith K, Iacobelli M, Saraf S, Welding K, Cohen J	USA, Canada, Brazil, Ethiopia, Uganda, Senegal, Niger, Mauritania, EU (28 Member States), Moldova, Turkey and Singapore	Global systematic review	• Banning tobacco sale or one or more of tobacco products
The Khan review Making smoking obsolete	Khan J.	United Kingdom	Policy review	• Banning tobacco sale in specific retail outlets
				• Banning tobacco sale or one or more of tobacco products
**Papers identified theough the database research**				
Theoretical impacts of a range of major tobacco retail outlet reduction interventions: modeling results in a country with a smoke-free nation goal	Pearson A, van der Deen F, Wilson N, Cobiac L, Blakely T	New Zealand	Cross-sectional	• Banning tobacco sale in specific retail outlets
				• Banning tobacco retail outlets in or within a minimum distance from specific facilities
Vatican beats Italy 1-0 in the tobacco endgame	Gallus S, Cattaruzza M, Gorini G, Faggiano F	Italy	Commentary (communication piece)	• Banning tobacco sale or one or more of tobacco products
Tobacco retail policy landscape: a longitudinal survey of US states	Luke D, Sorg A, Combs T, Robichaux C, Moreland-Russell S, Ribisl K, Henriksen L	United States	Longitudinal study	• Limiting the number of hours or days in which tobacco can be sold
				• Requiring a license to sell tobacco
				• Banning tobacco retail outlets in or within a minimum distance from specific facilities
				• Restricting tobacco retail outlets per geographic area
				• Requiring a minimum distance between tobacco retailers
				• Banning tobacco sale in specific retail outlets
Banning tobacco sales and advertisements near educational institutions may reduce students' tobacco use risk: evidence from Mumbai, India	Mistry R, Pednekar M, Pimple S, Gupta P, McCarthy W, Raute L, Patel M, Shastri S	Mumbai, India	Cross-sectional	• Banning tobacco retail outlets in or within a minimum distance from specific facilities
Ending tobacco sales in pharmacies: A qualitative study	Jin Y, Berman M, Klein E, Foraker R, Lu B, Ferketich A	United States	Qualitative study	• Banning tobacco sale in specific retail outlets
A Comprehensive Review of State Laws Governing Internet and Other Delivery Sales of Cigarettes in the USA	Chriqui J, Ribisl K, Wallace R, Williams R, O'Connor J, Arculli R. A	United States	Systematic review	• Banning home delivery of tobacco
Altering the availability or proximity of food, alcohol, and tobacco products to change their selection and consumption	Hollands G, Carter P, Anwer S, King S, Jebb S, Ogilvie D, Shemilt I, Higgins J, Marteau T	High-income countries (predominantely USA)	Systematic review	• Reducing tobacco products availability and proximity within a retail outlet
Four policies to end the sale of cigarettes and smoking tobacco in New Zealand by 2020	Laugesen M, Glover M, Fraser T, McCormick R, Scott J	New Zealand	Policy review	• Banning tobacco sale or one or more of tobacco products

### 2.5. Synthesis

After the phase of identifying policies that limit tobacco availability by regulating retail environment as per Tables 1–4, similar policies were compilied and policies were categorized to either covered by the WHO FCTC (mainly for other purposes than limiting the availability), and non WHO FCTC policies. This synthesis led to a total of sixteen policies identified that are summarized in Table 5 and will be discussed below.

**Table 5:** All policies identified.

**1. WHO FCTC policies**
1.1. Requiring a license to sell tobacco 1.2. Banning tobacco sale *via* vending machines 1.3. Promoting economically alternative activities to individual sellers 1.4. Banning ways of sale that constitute a way of advertising, promotion, and sponsorships
**2. Non-WHO FCTC policies**
2.1. Restricting tobacco retail outlets per density of population 2.2. Banning home delivery of tobacco 2.3. Banning tray/mobile tobacco sale 2.4. Capping the tobacco amount allowed per purchase 2.5. Requiring a minimum distance between tobacco retailers 2.6. Banning tobacco retail outlets in or within a minimum distance from specific facilities 2.7. Banning tobacco sale in specific retail outlets 2.8. Restricting tobacco retail outlets per geographic area 2.9. Government controlled outlets 2.10. Limiting the number of hours or days in which tobacco can be sold 2.11. Reducing tobacco products availability and proximity within a retail outlet 2.12. Banning tobacco sale or one or more of tobacco products

## 3. Results

### 3.1. WHO FCTC policies

#### 3.1.1. Requiring a license to sell tobacco

Article 15 of the WHO FCTC is mainly about policies to fight illicit trade in tobacco products. However, it flags the need for licensing to prevent illicit trade. The licensing requirement was further developed and explained in Article 6 of the Protocol to Eliminate Illicit Trade in Tobacco Products (3). Such policy could be a starting point to assess the density of tobacco selling points and then regulating them. The CTFK database shows that 52 countries have specific retail license to sell tobacco products (12). Out of the 31 countries responded to the survey, 13 countries reported that they have policies to revoke the license or to apply a fine in case of license condition violations (13).

There is evidence to suggest that high license fees will lead to decrease in number of tobacco retailers. A study done in Australia confirms this, estimating a 25% decrease in retailers (14). Making licensing of tobacco retailers a policy and identifying a cap on the number of licenses would give the license a higher commercial value that will promote compliance to retail regulations as violation would risk losing that asset. Losing the license as a penalty could be also used as a way to decrease number of retailers (5). The policies of establishing and increasing license fees are adopted in many cities in the United States (15). Licensing requirements and strict conditions to be met to get a tobacco retail license, in addition to innovative practices in tobacco licensing, are discussed in policy documents (16).

#### 3.1.2. Banning tobacco sale *via* vending machines

Article 16 of the WHO FCTC is mainly about protecting minors from having access to tobacco products. It requires the implementation of policies that ensure no access to vending machines by minors (3). The Article also requires prohibiting the introduction of tobacco vending machines to a total ban on tobacco vending machines. The guidelines for implementation of Article 13 of the WHO FCTC also require banning vending machines as they constitute, by their presence, a means of advertising and promotion of tobacco products (17). The CTFK database shows that 86 countries ban sale of tobacco products *via* vending machines, while 20 countries have some restrictions on vending machines that varies from banning them in places where minors are usually present to allowing them only in specific places such as hotels (12).

#### 3.1.3. Promoting economically alternative activities to individual sellers

Article 17 of the WHO FCTC states that “Parties shall, in cooperation with each other and with competent international and regional intergovernmental organizations, promote, as appropriate, economically viable alternatives for tobacco workers, growers and, as the case may be, individual sellers” (3). The relevant practice to this Article so far is mainly about alternative solutions to tobacco growers. However, in Kenya's tobacco control act, individual sellers can be understood as sellers at retail environment as the law states “The Government through the relevant ministries shall put in place policies to promote, as appropriate, economically viable alternatives for tobacco workers, distributors, retailers and individual sellers” (18).

Introducing incentives that encourage retailers to stop selling tobacco, such as a subsidized program to help them sell more fresh fruit and vegetables, is a suggested policy to reduce tobacco retail density (4). A successful example to such policy is passing the San Francisco‘s Tobacco Retail Density Policy after the adoption of the Healthy Retail San Francisco ordinance which helped corner stores in shifting to the business of fresh and healthy affordable food. Such program secured an opportunity to find common ground with the retail association and led to a density policy solution that was supported by all stakeholders (19).

#### 3.1.4. Banning ways of sale that constitute a way of advertising, promotion, and sponsorships

Article 13 of the WHO FCTC is mainly about banning tobacco advertising, promotion, and sponsorships. In its Guidelines for implementation (17), some regulations of retail environment were adopted. Although these policies aim mainly to reduce the demand on tobacco, the authors believe that they can also reduce tobacco supply within the retail environment. These regulations include:

– Banning internet sale of tobacco. The CTFK database shows that 63 countries ban sale of tobacco products *via* the internet (12).
– Banning tobacco sale at educational establishments or at hospitality, sporting, entertainment, music, dance and social venues or events. The CTFK database shows that 76 countries ban tobacco sale in schools/educational facilities, 45 countries ban tobacco sale in stadiums/arenas, 31 countries ban tobacco sale in cultural facilities, and 26 countries ban tobacco sale in playgrounds (12).

### 3.2. Non-WHO FCTC policies

#### 3.2.1. Restricting tobacco retail outlets per density of population

Possible models to decrease tobacco retail density could include a model where a restricted number of licenses is based on an agreed number of tobacco retail outlets per 100,000 population, and such licenses could be auctioned to the highest bidder (5). A population-based retailer caps is implemented in Hungary allowing only one store for every 2,000 residents (20).

France flagged 3,500 inhabitants as the number commonly required for opening a tobacconist shop (21). In South Korea, density of population does not limit the number of tobacco sellers, but local officials consider density of population in setting the criteria for certain distance between tobacco sellers (22). In Italy, municipalities with no more than 10,000 inhabitants are allowed to have one tobacco shop every 1,500 inhabitants, but there is also a policy that requires minimal distance between two tobacco sellers as per inhabitants' density. This required distance is 200 meters in cities with more than 100,000 inhabitants, 250 meters in cities with more than 30,000 inhabitants, and 300 meters in cities up to 30,000 inhabitants (23).

#### 3.2.2. Banning home delivery of tobacco

Although banning tobacco internet sale is a requirement under the WHO FCTC, some countries expanded this to ban all types of distal sale and tobacco home delivery. Costa Rica prohibited tobacco sales to the consumer by telephone, digital, electronic, internet, mail, and other means (24). South Korea banned retailers from selling tobacco to consumers by way of postal sale or electronic transactions (22). Spain banned home delivery of tobacco products. Belgium banned all types of distal sales of tobacco (13). A review of state laws in the United States found that five states banned direct-to-consumer shipment of cigarettes (25).

#### 3.2.3. Banning tray/mobile tobacco sale

A couple of countries banned tray/mobile sale of tobacco products. In Guyana no person shall go into any public place carrying any tobacco product, electronic delivery system, or component, in a tray, container or otherwise for the purpose of making sales or commercially displaying the product (26). Ireland prohibited the sale of tobacco products from mobile units/containers, from temporary or movable premises (27).

#### 3.2.4. Capping the tobacco amount allowed per purchase

Capping amount allowed per purchase is implemented on other products. For example, in some settings the sale of paracetamol tablets is restricted by law to a maximum pack of 16 tablets without the supervision of a pharmacist (28). This is not the case for tobacco. Although many countries put a cap on the amount of tobacco to be purchased from duty free zones such as airports, this policy was not extended to be applied in regular retail environment. A review suggested restricting the amount of tobacco smokers could purchase over a given time to promote a gradual decrease in tobacco-use till quitting, this could happen by introducing an upper weekly limit to tobacco product purchases (5).

A couple of countries apply such policies but with very high amount allowed. Andorra prohibits the sale to individuals for an amount higher than 10,000 cigarettes. In the case of cigars the ban is for quantities exceeding 5,000 units (cigars or cigarettes <3 gram per piece) or 2,500 units (cigars or cigarettes <3 gram per piece). For waterpipe tobacco and smoking tobacco, the sale of more than ten kilograms is prohibited, these restrictions apply per sale and per person (29). In Spain, although there is no restriction on the allowed amount per purchase, the sale of tobacco products must be accompanied by their corresponding invoice or sale if the quantity is more than 800 cigarettes, 200 units in the case of cigars, 400 units in the case of cigarillos, or one kilogram of other tobacco products (30).

#### 3.2.5. Requiring a minimum distance between tobacco retailers

A policy document suggested limiting the proximity of tobacco retailers to each other, with retailers not being allowed to sell tobacco products within 1,000 meters of another tobacco retailer (16). Having minimum required distance between tobacco retailers prevents clustering of tobacco outlets in certain areas such as economically disadvantaged districts (20). A study in the United States revealed that regulating the minimum allowable distance to 500 feet between tobacco outlets, if implemented, would reduce tobacco retailers' density by 22.1% (31).

Requiring certain distance between tobacco retailers is discussed in many of the United States cities and passed in a couple of them (15). California bans tobacco retailers from opening new stores within 200 feet of another store, that goes up to 500 feet in the unincorporated parts (20). The tobacco retailers density policy in San Francisco applied a standard of no tobacco sale permitted within 500 feet of another location permitted to sell tobacco (19). In South Korea, although the number of tobacco retail outlets per unit area is not limited, a certain distance must be maintained between retailers that differs according to criteria from one region to another (22). Spain has a policy that requires at least 150 meters distance between tobacco selling shops, and grants complementary status for tobacconists opening in rural zones (30).

#### 3.2.6. Banning tobacco retail outlets in or within a minimum distance from specific facilities

The Guidelines for implementation of the WHO FCTC require banning tobacco vending machines; internet sale of tobacco; and tobacco sale at educational establishments or at hospitality, sporting, entertainment, music, dance and social venues or events. Some countries expanded this in their polices to either ban tobacco sale in other facilities or to ban tobacco sale within a minimum distance from specific facilities. CTFK database shows that 65 countries ban tobacco sale in healthcare facilities (12). Portugal banned tobacco sale in many facilities including covered car parks and in the enclosures of automatic cash withdrawals (32). France banned tobacco sale in protected areas that includes perimeter of health, education and sports facilities or training, collective accommodation, and leisure establishments for young people (33).

A policy document suggested the establishment of safe routes policy by banning tobacco sale in designated access routes to schools, in addition to identifying specific distance from schools and youth-oriented facilities where tobacco sale is prohibited. The document suggested tobacco retailers should be at least 500 meters away from schools, community centers, sport or leisure facilities (16). A study in the United States revealed that restricting sales of tobacco products within 1,000 feet of schools, if implemented, would reduce tobacco retailers' density by 17.8% (31). A study in New Zealand suggested that elimination of outlets within 2 km of schools yielded an estimated lower smoking prevalence compared to no intervention (34).

Many places already banned tobacco sales near youth-populated areas (20). Prohibiting tobacco sales in locations where youth frequent are adopted in many cities in the United States (15). A number of countries banned tobacco sale from different facilities such as health, education, sports, childcare, religious, government, public, cultural and leisure facilities. The minimum distance required is varied such as 10 meters in Saint Lucia, 50 meters in Georgia, 100 meters in Chile and Honduras, 500 feet in San Francisco, 500 meters in Saudi Arabia and Guatemala, and 1,000 meters in Qatar (12, 13, 19).

#### 3.2.7. Banning tobacco sale in specific retail outlets

Some countries banned tobacco selling in specific retail outlets. Czech Republic prohibits the sale of tobacco in food stores, catering establishments, and refreshment stands (35). A tobacco retailers density policy in San Francisco banned tobacco sales in restaurants, bars, or other tobacco shops that are not already permitted (19). A study in New Zealand suggested that permitting tobacco sales at only 50% of liquor stores resulted in large cost increase of getting tobacco (~$60/pack in rural areas) and yielded an estimated lower smoking prevalence than with no intervention (34). A recent review and policy document in the United Kingdom suggested banning tobacco sale in supermarkets (36).

Prohibiting sales in specific venues such as pharmacies is adopted in a number of places considering the conflict of interest for pharmacies to sell tobacco while offering medicine for tobacco-related diseases (20). Banning tobacco sales in pharmacies is a sensible public health policy with a proven positive impact of reducing tobacco sales density and smoking prevalence (37). This policy has been discussed in many of the United States cities (15). A study in North Carolina revealed that prohibiting sales of tobacco products in pharmacies or stores with a pharmacy counter, if implemented, would reduce tobacco retailers' density by 13.9% (31). A study in California and Massachusetts suggested that the process of adopting the tobacco-free pharmacy laws was smooth, with a few barriers (38).

Communities can also require that tobacco be sold solely by tobacco-only retailers as a method to control and decrease number of outlets selling tobacco (20). France allows only tobacconists to sell tobacco and it bans a shop manager to apply for a license to open another one. France also prohibits the sale of tobacco in shopping centers and shopping malls next to supermarkets of more than 1,000 square meters. Saudi Arabia allows tobacco to be sold only in supermarkets not <100 square meters. Spain allows tobacco selling only to tobacconists or vending machines. Netherlands plan to ban tobacco sale in supermarkets by 2024, and to make tobacco sold only in tobacco specialty stores by 2030 (13).

#### 3.2.8. Restricting tobacco retail outlets per geographic area

A policy to reduce number of retailers in a defined geographic area is the cap and winnow approach, which involves setting a limit at the number of existing tobacco retailers, then allowing lower number of new outlets than those who have failed to renew their licenses or have them revoked (20). A policy document suggested prohibiting tobacco retailers from locating in residential zones, and restricting the location of tobacco retailers to particular zones in a community (16).

Restricting retailers in certain zones such as residential zones is a policy discussed in many of the United States cities and passed in a couple of them. Limiting or capping the total number of licenses in a specific area is a policy discussed in many United States cities (15). A tobacco retailers density policy in San Francisco caps the number of tobacco sale permits in each of the City's 11 Supervisorial Districts at 45, limiting the citywide total to 495. The policy also added that tobacco sale permits will not be issued in locations that have never had a tobacco license in the past (19).

#### 3.2.9. Government controlled outlets

Possible models to decrease tobacco retail density could include the nationalization of tobacco retailing involving a single network of government-controlled outlets (5). A suggested policy option in the literature is to restrict tobacco sales to a limited number of controlled outlets. A similar model has been used for the sale of alcoholic beverages before (16). A transition to government operated stores could solve the current contradiction between acknowledging and raising awareness of tobacco harms, while still allowing its sale. Such transition could allow governments to enforce better compliance to tobacco control policies and will provide governments with the tool to better regulate tobacco products and to move forward toward limiting tobacco availability (39).

#### 3.2.10. Limiting the number of hours or days in which tobacco can be sold

A study in the United States discussed the planned and existing policies relevant to tobacco retail policy. It identified that a policy to limit the number of hours or days in which tobacco can be sold was planned/proposed twice in 2014 (15).

#### 3.2.11. Reducing tobacco products availability and proximity within a retail outlet

The idea of altering the availability and proximity of tobacco products within a retail outlet was raised in a review about effects of availability or proximity of food, alcohol, and tobacco products to change their selection and consumption. The review didn't include studies particularly on tobacco, but the interventions mentioned included decreasing tobacco availability by providing a reduced range of types of tobacco product and making a lesser amount of cigarettes available in a shop. The intervention to decrease tobacco proximity was by moving tobacco products farther away from people to alter the degree of convenience and effort required for potential consumers to select or consume these products (40).

#### 3.2.12. Banning tobacco sale or one or more of tobacco products

The scope of this review is about the retail environment regulations that affects all types of tobacco and not a certain targeted policy or endgame strategies, but its worth highlighting that some countries ban sale of all or certain types of tobacco. Endgame theories suggested different ways for phasing out tobacco products, such as allocating national sales quotas per manufacturer or importer, with annual 5% reduction of the allocated amount, and then be reduced by 5% every 6 months (41). A recent policy review in the United Kingdom suggested freezing the tobacco market and banning the introduction of any new tobacco products to make the market stagnant and to avoid the presence of new available or attractive tobacco products (36).

The CTFK database shows that 12 countries banned waterpipe sales, and 18 countries banned the sale of smokeless tobacco products (12). According to a recent review, 40 countries have active or pending policies that range from banning flavored tobacco, to banning flavor descriptors and images on packaging (42). In the Vatican, all sales of tobacco products were banned, a decision that was well received as a positive step toward counteracting practices that are harmful to the health of citizens (43). There is also evidence of the tobacco industry's opposition to such policies (44).

## 4. Discussion

### 4.1. Identified policies

This review identified 16 policies to reduce tobacco availability by regulating tobacco retail environment. The data synthesis and results' presentation followed the categorization of policies to either WHO FCTC polices or non-WHO FCTC policies because of the huge importance of the treaty in guiding the global performance in tobacco control. The identified policies can however be categorized to four main themes: policies that limit the number of tobacco retailers, policies that limit the ways of tobacco sales, policies that limit accessibility to tobacco products when they are available in the retail environment, and policies that ban tobacco sales.

Policies that limit the number of tobacco retailers included three policies covered by the WHO FCTC and six policies not covered by the treaty. The ones covered by the treaty are requiring a license to sell tobacco; promoting economically alternative activities to individual sellers; and banning tobacco sale at educational establishments or at hospitality, sporting, entertainment, music, dance and social venues or events. The ones that were not covered by the treaty are restricting tobacco retail outlets per density of population; requiring a minimum distance between tobacco retailers; banning tobacco retail outlets in or within a minimum distance from specific facilities; banning tobacco sale in specific retail outlets; restricting tobacco retail outlets per geographic area; and selling tobacco only in government-controlled outlets to potentially limit the number of outlets.

Policies that limit the ways of tobacco sales included two policies covered by the WHO FCTC and two policies not covered by the treaty. The ones covered by the treaty are banning tobacco sale *via* vending machines and banning internet sale of tobacco. The ones that were not covered by the treaty are banning home delivery of tobacco and banning tray/mobile tobacco sale.

Policies that limit accessibility to tobacco products when they are available in the retail environment were not covered by the WHO FCTC. These policies are mainly three ones: capping the tobacco amount allowed per purchase; limiting the number of hours or days in which tobacco can be sold; and reducing tobacco products availability and proximity within a retail outlet.

Policies that ban tobacco sales included banning tobacco sale or one or more of tobacco products. This policy is listed under non-WHO FCTC policies, but it is worth flagging that the treaty decisions refer to banning flavored tobacco.

### 4.2. Importance of the topic

A global review documented that points of sale are used for tobacco advertisement even for children and youth by displaying of cigarettes near snacks, sweets and sugary drinks; placement of cigarette advertisements near the eye-level of children; advertisements and display of flavored cigarettes; and sale of single sticks of cigarettes (45). In Indonesia, a study about cigarette retailer density around schools and neighborhoods found that around 9.7% of the schools in Denpasar have at least one cigarette seller within a 25 meter radius and 96.8% within a 250 meter radius (46). A study done in two cities in India found that around 20% of tobacco vendors were observed operating within 100 yards of a school, with an average three or four tobacco vendors operating within 100 yards of each school (47). A study in India suggested that a tobacco sales ban near educational institutions could be expanded beyond 100 meters (48).

### 4.3. Tobacco industry position regarding retail environment

In 2020, the tobacco industry spent a million dollars every hour in the United States to make its presence known in the retail environment, with a total of $8.2 billion spent over the year (49). A study in Australia revealed that tobacco industry gave retailers cash and paid vacations with the objective of increasing market share and driving sales (50). Furthermore, it has also been documented that tobacco companies target retailers through competitive discounts, cash payments, prizes and gifts aimed at building positive relationships with them (51, 52). This relation allows the tobacco industry to influence the merchandising of tobacco products, which ultimately influences the sale and promotion of tobacco (51–53).

Tobacco industry gives particular interest to its relationships with tobacco retailers. A recent scoping review concluded that tobacco industry-retailers' agreements for pricing discounts and prime placement of products and advertising are prevalent around the world. Such agreements allow the tobacco industry to promote its products and undermine tobacco control efforts in the retail setting. The review recommended banning such agreements. The importance of retailers' compliance to implementing tobacco control policies, in addition to the aim of limiting tobacco availability raises the idea of government-controlled outlets as a potential solution (54).

Tobacco companies oppose retail reduction policies, and in one letter sent by Japan Tobacco International to the Jordanian government, the company opposed the policy of declining to license tobacco sales within 200 meters from residency areas, mosques, educational facilities, and health facilities. The company stated in the letter that such policy equals in its severity a complete ban on tobacco sales. The company also opposed any restrictions on size or number of areas allocated to tobacco sales in commercial centers (55). It is reported in the media that the tobacco industry works to increase its presence in stores by increasing its field force (56).

### 4.4. Effectivness of policies

Studies show the effects of regulation of the retail environment in influencing overall tobacco purchases, and there is strong evidence that having fewer retails reduces the level of impulse purchasing of cigarettes and tobacco goods (4). For example, a Canadian study found that one-third of smokers would smoke less if they had to travel further to buy cigarettes, especially younger smokers (57). Similarly, another study in Australia found that even in the absence of tobacco products at the checkout counter, just the sight of tobacco retail outlets prompted impulse purchases (58, 59).

Policy analysis shows that reducing the convenience of obtaining tobacco products increases the cost to the smoker including the time, effort, and money spent to obtain tobacco. Therefore, policies that limit availability of tobacco will clearly affect the convenience of obtaining tobacco (16). A study in New Zealand suggested that with a law that required a 95% reduction in tobacco outlets, the cost of a pack of 20 cigarettes increased by 20% in rural areas and 10% elsewhere and yielded an estimated lower smoking prevalence compared to no intervention (34). A recent meta-analysis study concluded that decreased levels of tobacco density and proximity are associated with lower tobacco use (60).

### 4.5. Feasibility of policies

A study in the United States discussed the legal challenges for policies such as requiring minimum distance between retailers, limiting retailers in each geographic area, linking number of tobacco retailers to population size, and banning tobacco sales at or within a certain distance of certain places. The study concluded that courts are likely to reject constitutional challenges to carefully crafted laws that reduce the number of tobacco retailers (20).

This review shows that the policies covered by the WHO FCTC are more frequently implemented than the ones not covered by it. Overall, many policies of limiting tobacco availability by regulating tobacco retail environment are available. The WHO FCTC and its Conference of Parties decisions provide a great opportunity for scaling up effective strategies, and bringing them to the attention of tobacco control at a global level, especially with the growing evidence of both public and experts' support for relevant policies (61, 62).

### 4.6. Limitations of the review

The authors recognize the limitation of the study considering the difficulties in reaching WHO FCTC Focal Points in all Parties, and in reaching tobacco control officials in countries that are not Parties to the WHO FCTC. In addition, the survey shared with the WHO FCTC Focal Points was only in English so all other languages were excluded. Furthermore, many of the policy documents are believed to be unpublished online and so were not included in this review.

## 5. Conclusion

Evidence on the effectiveness of supply reduction overall is available. Policies of regulating tobacco retail environment to reduce tobacco availability are effective, feasible, and already implemented. The extent of their implementation differs, mainly that the ones covered by the WHO FCTC are more widely implemented.

This review flagged that a wide range of policies not covered by the WHO FCTC are implemented by countries, however not much research is conducted to assess such polices or to evaluate the process of their implementation. This review documented the countries innovation in terms of policies to reduce tobacco availability by regulationg retail environment with overall 12 polices not covered by the WHO FCTC. There is a potential for these policies to be scaled up at global levels as a theme for tobacco control, a crutial step to do that could be a decision from the WHO FCTC Conference of Partices to require research and implementation of such policies.

## Author contributions

RA conceptualized this review, gained the ethical approval, and collected the data through searching the WHO FCTC and Protocol documents, gray literature review, Focal Points survey study, and databases review and did the data analysis and synthesis and prepared the first draft of the manuscript. ZA did the initial database search and supported in the screening of databases results. MB supervised the implementation of this study and provided feedback during the manuscript preparation. All authors read and agreed on the final manuscript.
