# Supplementary material for: Policies regulating retail environment to reduce tobacco availability: A scoping review
Source: Front Public Health. 2023 Feb 14;11:975065. doi: 10.3389/fpubh.2023.975065 (PMC9971920; doi:10.3389/fpubh.2023.975065)
Supplement: Supplementary file 1 [file Data_Sheet_1.docx]

**Annex 1**

**Research project - Reducing tobacco supply through retail environment regulations**

…….

As part of the project, we are interested to learn whether, in your jurisdiction, any of the following are implemented or planned:

- Policies requiring the limitation of the number of tobacco retail outlets per Km^2^.
- Policies requiring limited number of outlets permitted to sell tobacco according to density of population.
- Policies requiring minimum distance of tobacco outlets from educational, sport and health facilities.
- Policies capping amount of tobacco allowed to be purchased over a specific period of time (per single purchase, for example).
- Policies banning tobacco home delivery.
- Policies banning self-serve tobacco vending machines.
- Policies banning specific outlets from selling tobacco products, such as banning sale in pharmacies or petrol stations.
- Policies requiring application to a specific licence for outlets to sell tobacco.
- Policies of retail licence loss in case of any violation to tobacco laws.
- Any other policies to limit access to/ availability of tobacco products through regulating the retail environment.
